# The role of piRNAs in predicting and prognosing in cancer: a focus on piRNA-823 (a systematic review and meta-analysis)

**DOI:** 10.1186/s12885-024-12180-2

**Published:** 2024-04-16

**Authors:** Mohammad Taghizadeh, Tohid Jafari-Koshki, Vahid Jafarlou, Mortaza Raeisi, Leila Alizadeh, Yousef Roosta, Somaieh Matin, Rahele Jabari, Daniel Sur, Abbas Karimi

**Affiliations:** 1 Department of Molecular Medicine, Faculty of Advanced Medical School, Tabriz University of Medical Sciences, Tabriz, 5166614756 Iran; 2 Department of Statistics and Epidemiology, Faculty of Health, Tabriz University of Medical Sciences, Tabriz, 5166616471 Iran; 3 Cancer Institute of Imam Khomeini Hospital, Tehran University of Medical Science, Tehran, 1419733141 Iran; 4 Hematology and Oncology Research Center, Tabriz University of Medical Sciences, Tabriz, 5166616471 Iran; 5 Gastroenterology and Liver Diseases Research Center, Tabriz University of Medical Sciences, Tabriz, 5166616471 Iran; 6 Department of Internal Medicine, School of Medicine, Urmia University of Medical Sciences, Urmia, 5714783734 Iran; 7 Solid Tumor Research Center, Cellular and Molecular Medicine Research Institute, Urmia University of Medical Sciences, Urmia, 5714783734 Iran; 8 Hematology, Immune Cell Therapy, and Stem Cells Transplantation Research Center, Clinical Research Institute, Urmia University of Medical Sciences, Urmia, 5714783734 Iran; 9 Department of Internal Medicine, School of Medicine, Ardabil University of Medical Sciences, Ardabil, 8599156189 Iran; 10 Department of Oncology, The Oncology Institute “Prof. Dr. Ion Chiricu¸tă”, Cluj-Napoca, 400015 Romania; 11 Department of Medical Oncology, The Oncology Institute “Prof. Dr. Ion Chiricu ¸t ˘a”, 400015 Str. Republicii 34-36, Cluj-Napoca, 400006 Romania; 12 Department of Nutrition Science, Faculty of Medical Science, Urmia University of Medical Science, Urmia, 5714783734 Iran; 13 Department of Molecular Medicine, Faculty of Advanced Medical Sciences, Tabriz University of Medical Sciences, Golgasht St., Tabriz, East Azerbaijan 5166614756 Iran

**Keywords:** piRNA, Cancer, Survival, Prognosis, Systematic review, Meta-analysis

## Abstract

**Introduction:**

This article examines the potential of using liquid biopsy with piRNAs to study cancer survival outcomes. While previous studies have explored the relationship between piRNA expression and cancer patient outcomes, a comprehensive investigation is still lacking. To address this gap, we conducted a systematic review and meta-analysis of existing literature.

**Methods:**

We searched major online databases up to February 2024 to identify articles reporting on the role of piRNA in cancer patient survival outcomes. Our meta-analysis used a random-effects model to pool hazard ratios with 95% confidence intervals (CI) and assess the prognostic value of deregulated piRNA-823. For survival analysis, the Kaplan–Meier method and COX analysis were used.

**Results:**

Out of 6104 articles screened, 20 met our inclusion criteria. Our analysis revealed that dysregulated piRNA expression is associated with cancer patient survival outcomes. Specifically, our meta-analysis found that overexpression of piR-823 is significantly linked with poorer overall survival in patients with colorectal cancer and renal cell cancer (HR: 3.82, 95% CI = [1.81, 8.04], I^2^ = 70%).

**Conclusion:**

Our findings suggest that various piRNAs may play a role in cancer survival outcomes and that piRNA-823 in particular holds promise as a prognostic biomarker for multiple human cancers.

**Implications for cancer survivors:**

Our systematic review and meta-analysis of piRNA-823 has important implications for cancer survivors. Our findings suggest that piRNA-823 can be used as a prognostic biomarker for predicting cancer recurrence and survival rates. This information can help clinicians develop personalized treatment plans for cancer survivors, which can improve their quality of life and reduce the risk of recurrence.

## Introduction

Cancer is a complex disease that is influenced by multiple factors, and its intricate pathology poses challenges to the prevention, diagnosis, treatment, and survival of the disease [1]. Although significant progress has been made in cancer diagnosis, such as Next-Generation sequencing (NGS) [2] and treatment options like neoadjuvant chemotherapy (NAC), targeted therapy, and gene therapy. The complete treatment of cancer remains a significant challenge. Despite these advancements, there are still numerous obstacles to overcome before achieving a cure for all types of cancer [3]. Early detection of cancer is vital as it significantly increases survival rates. Unfortunately, nearly half of all cancers are diagnosed at an advanced stage, which can limit treatment options and reduce the chances of cure [4].

Recent studies have shown that liquid biopsy is a highly promising and non-invasive approach for cancer diagnosis and monitoring [5–7]. This technique offers minimal invasiveness and repeated sampling, making it an attractive option for monitoring tumor occurrence and recurrence in real-time, as well as evaluating prognosis and treatment response [8]. In addition, it could be a valuable tool for advancing early detection efforts [9]. The idea of a liquid biopsy involves various methods used to find biomarkers in the body fluids like blood, plasma/serum, urine, CSF, saliva, ascites, or pleural effusion of people with cancer [10, 11]. The analysis of liquid biopsy is a growing area in translational cancer research with the potential to transform cancer treatment. Liquid biopsies can identify a range of circulating tumor products, including circulating tumor cells (CTCs), circulating tumor DNA (ctDNA), circulating messenger RNA (mRNA), circulating non-coding RNA, circulating extracellular vesicles (EVs), and tumor-educated platelets (TEPs) [10, 12].

Among the various biomarkers that can be detected by liquid biopsy, Piwi-interacting RNAs (piRNAs) are a novel class of small non-coding RNAs that play a crucial role in maintaining genome stability [13, 14]. They are typically 24–31 nucleotides in length and interact with PIWI proteins to form the piRNA silencing complex (piRISC) [15]. piRNAs are found in highly conserved clusters throughout the genome, but they are only present at a limited number of loci [16, 17]. Similar to miRNAs and siRNAs, the complexes of piRNAs and Piwi proteins may be involved in post-transcriptional gene silencing [18]. Particularly in the silencing of retrotransposons, this is partly because most piRNAs are antisense to retrotransposon sequences [19].

New research has demonstrated that piRNAs are expressed aberrantly in various cancer cell types and contribute to the development, progression, metastasis, and resistance to treatment of tumors [20]. Despite this, the potential clinical significance of their role is not yet fully understood [21]. There are several types of piRNAs that affect cancers [22, 23]. For example, piR-54265 is an oncogenic piRNA in colorectal cancer (CRC) and has elucidated its underlying molecular mechanism for driving malignant phenotypes in CRC cells [24]. piR-651 is associated with the progression of non-small cell lung carcinoma (NSCLC). A study found that high expression of piR-651 was associated with a higher risk of death in patients with NSCLC [25]. piR-823 is down-regulated in tumor tissue but positively correlated with worse outcomes, indicating its complex role in renal cell carcinoma (RCC) pathogenesis [26].

Among the piRNAs extensively investigated in cancer research, piRNA-823 stands out as a prominent subject. Recent researches indicate that increased expression of piRNA-823 is linked to the survival outcomes of cancer patients [26, 27]. Although there are many related records on the role of piRNA-823 in cancers, these records are scattered and lack systematic organization and summary. Therefore, this study summarizes and analyzes the role of expression patterns of piRNA-823 and the prognosis of cancer patients.

Based on the various roles of piRNAs in different types of cancer, to date, there is no literature that evaluates the correlation between dysregulation of piRNA expression levels and cancer prognosis. The results of some studies are inconsistent, and a single study may have insufficient data. Therefore, the objective of this systematic review and meta-analysis is to assess the impact of dysregulated expression of piRNAs on the survival outcome of cancer patients. Specifically, we will focus on the role of piRNA-823 and its prognostic significance for patient survival across different types of cancers. Our goal is to clarify the clinical relevance of piRNA-823 as a potential biomarker for cancer diagnosis and prognosis and to provide insights for future research in this area.

## Methods

### Systematic review

This report follows the PRISMA 2020 structure.

### Eligibility criteria

We examined the correlation between the role of piRNAs and any of the following types of survival analysis: Overall/cumulative survival (OS), Progression-free survival (PFS), Disease-free survival (DFS), Recurrence-free survival (RFS), Event-free survival (EFS), and Metastasis-free survival (MFS). OS measures the time from diagnosis to death from any cause [28]; PFS measures the time from first treatment to the identification of cancer progression or death from any cause [28]; DFS measures of time after treatment during which no sign of cancer is found [29]; RFS measures the time from cancer cure to the identification of cancer progression/recurrence [30]; EFS measures of time after treatment that a group of people in a clinical trial has not had cancer come back or get worse [31]; and finally MFS measures the time from diagnosis to the identification of a metastatic event [32].

We utilized MeSH terms and added extra search terms to broaden the scope of our search and include a wider range of relevant studies. This approach was intended to make our analysis more comprehensive and ensure that we capture all relevant literature on the topic.

### Exclusion criteria

We implemented the following criteria to exclude articles from our analysis: (i) articles that were not accessible in full-text electronically; (ii) articles published in languages other than English; (iii) comments, letters, editorials, protocols, guidelines, case reports, and review articles; (iv) in vitro or preclinical studies; (v) studies lacking sufficient outcome data; (vi) studies that solely investigated genetic alterations of piRNAs, such as polymorphisms or methylation patterns.

We retained studies that reported hazard ratios (HR) and standard error (SE) or confidence interval (CI) or provided life tables for comparing two groups of high- and low-expression levels of various piRNAs [33, 34]. Hence, those studies with more than two groups (e.g. comparing quartiles of expression levels) were excluded from review and meta-analysis [35, 36].

### Inclusion criteria

Our inclusion criteria for articles were as follows: (i) studies that examined piRNA expression among cancer patients and control groups; (ii) studies that included data on survival outcomes of patients; (iii) any study that involved quantitative analysis of piRNAs using methods such as quantitative PCR (qPCR), in situ hybridization (ISH), microarray, or sequencing was eligible for inclusion; iii) Studies with sufficient data to generate HR and 95% CI or Kaplan–Meier curves, and (iv) studies published in the English language.

### Information sources

We conducted a systematic search of four databases (PubMed, Scopus, Web of Science, and Wiley Online Library) spanning 8 years from 2015 to 2024. Two independent authors (MT and AK) performed the search to identify potentially eligible articles published in the English language. Our analysis focused on investigating the associations between expression levels of piRNAs and prognosis in human cancer. We used a combination of MeSH terms and subsequent keywords to refine our search strategy., including (“Overall Survival”, “Disease-Free Survival”, “Progression-Free Survival”, “Recurrence-free survival”, “Event-free Survival”, “Survival Analysis”, “Kaplan–Meier Estimate”) AND (“piRNA”, “piRNAs”, “PIWI-interacting ribonucleic acid”, “PIWI-interacting RNA”) AND (“cancer”, “carcinoma”, “tumor”, “neoplas*”, “tumor”, “malignan*”, “metastat*”, “metastas*”, “leukemia”, “leukemia”, “lymphoma”) were used to search the literature. Moreover, search terms that had no available MeSH terms included (“Liquid Biopsy Analysis”, “Prognostic Value”, “Prognostic Factor”, “Prognostic Indicator”, “Prognostic and Predictive Biomarkers”, “Recurrence Risk”, “Predictive for Outcome”, “recurrence*”,). The search was last updated to include articles published through February 1, 2024.

### Study selection

We used the EndNote 21 to remove duplicate records. We screened the titles and abstracts of articles to identify those that were relevant to our study. The full manuscript of each relevant article was then screened against our eligibility criteria. Any uncertainties were resolved. by two of the authors (MT and AK) The data was collected and saved in Microsoft Excel Spreadsheet Software 2021. If the information was unclear or confusing, like when a study mentioned different piRNA quantification methods without specifying which one was used for the survival analysis, we moved it to a separate sheet and marked it as "unclear." To ensure thorough and transparent data collection, we gathered the following details from each study: basic study information such as the title, authors, publication year, number of patients, piRNA quantification methods, follow-up duration, cancer type, and journal of publication. We also collected additional details from eligible articles, including the study cohort's country of origin, sample size, and the total number of piRNAs analyzed.

### Data synthesis and statistical analysis

In this study, the survival outcome was determined by utilizing data from various metrics to characterize the findings, including HR, Relative Risk (RR), Odds Ratio (OR), *p*-value, or Cox Regression, the type of analysis conducted (Univariable or Multivariable), the SE, and the number of censored participants during the follow-up period. HRs were aggregated using I^2^ statistics to assess the heterogeneity among the relevant studies.

We conducted a meta-analysis on piRNA-823 to evaluate univariate OS. SE was calculated using the formula (upper limit of CI/lower limit of CI) / (2 × 1.96). Estimates were synthesized using a random-effects model and estimated via the restricted maximum-likelihood ratio method. Heterogeneity was evaluated using Q and I^2^ statistics, with the 95% CI of I^2^ also computed [37, 38].

To calculate the effect size and 95% CI, it was imperative for the included studies to provide adequate information. Studies were required to report the HR and its 95% CI or present survival curves that could be digitized using GetData Graph Digitizer software to extract the HR value. In cases where articles only presented survival curves without HR and its 95% CI, digitization was conducted to obtain the necessary data. Forest plots were utilized to display the meta-analysis outcomes, with these analyses performed using Review Manager (RevMan) [39] and for publication bias used Comprehensive Meta-Analysis (CMA) [40]. Data for each type of piRNA were pooled irrespective of cancer type.

## Results

### Literature search and description of studies

**Fig. 1:**
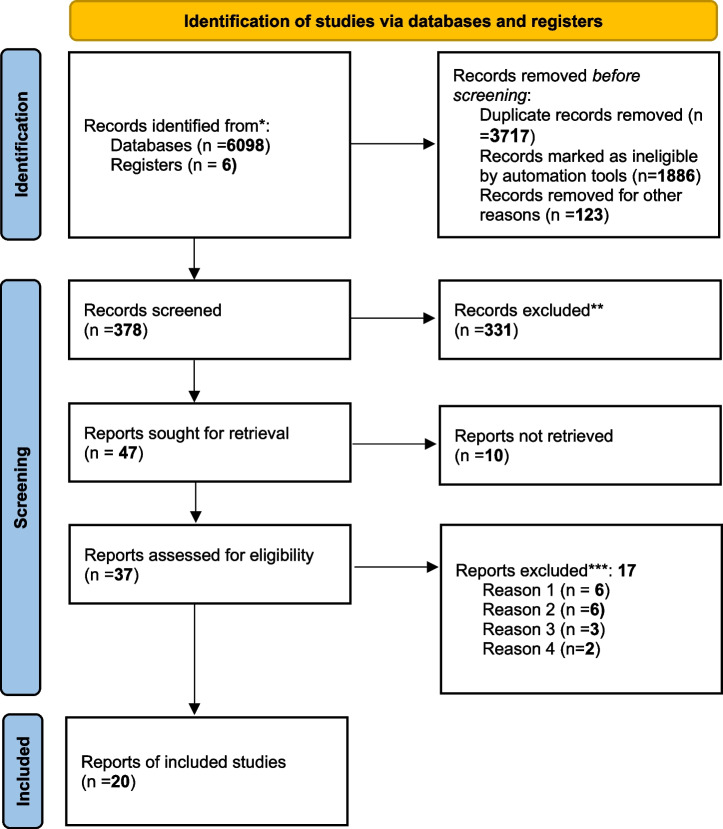
In this study, we utilized the ‘PRISMA 2020 flow diagram’ template, which is a visual representation of the flow of information through the different phases of a systematic review. The template includes searches of only databases and registers. Please refer to the PRISMA flow diagram of the study for more details * The trial registers including clinicaltrials.gov and trial search.who.int/AdvSearch.aspx were used in our search strategies ** The titles and abstracts of articles are not relevant to our research questions *** Reasons: Reason 1. There were no K-M survival curves indicating the specific event/endpoints Reason 2. The risk sets in time intervals were not included in the HR (Hazard ratio). Also, asking the authors for raw data was not successful Reason 3. Animal experiment included Reason 4. Not available data

The flowchart illustrates the search and selection strategy employed in the study (Fig. 1). We performed a systematic search for studies that investigated the potential role of piRNAs in the prognostic significance of cancer patients and identified 6,104 records using a detailed list of search terms (Fig. 1). After applying the exclusion criteria, a total of 1886 studies were excluded, resulting in 378 articles selected for further evaluation. Furthermore, 331 articles were excluded because of title and abstract screening criteria. Then 47 records remained eligible for full-text assessment. After full-text assessments, 20 studies meeting the search criteria were eligible for final review. Tables 1 and 2 provide an overview of the studies having eligible time-to-event analysis data and their main clinical characteristics.

**Table 1:** The main characteristics of the included studies

No.	Authors and years	Country	piRNA type(s)	piRNA(s) quantification	No. of patients	No. of control	Analysis method	Hazard ratio (HR) (95%CI)	Median follow-up months	Ref.
1	Huiying Han, et al. (2021)	China	piRNA-30473	Microarray analysis	42	N/A	Univariate and multivariate	Univariate analysis: HR = 2.193836	50	[41]
								Multivariate analysis: HR = 1.9892		
2	Guoqing Li, et al (2023)	China	piRNA-25783	ISH	15	N/A	Not reported	HR = 1.006	72	[42]
3	Lisha Ai et al (2019)	China	piRNA-823	qRT-PCR	72	N/A	Not reported	HR = 2.50	40	[43]
4	Robert Iliev, et al (2016)	Czech Republic	piRNA-823	qRT-PCR	472	116	Not reported	HR = 1.024	150	[26]
5	Jonas Busch, et al (2015)	Germany	piRNA-30924, piRNA-57125, and piRNA-38756	Microarray analysis	106	77	Univariate and multivariate	Univariate analysis: piRNA-30924 HR = 2.90, piRNA-57125 HR = 0.30, and piRNA-38756 HR = 3.96	120	[44]
								Multivariate analysis: piRNA-30924 HR = 2.04, piRNA-57125 HR = 0.50, and piRNA-38756 HR = 1.93		
6	Ailin Qu, et al (2019)	China	piRNA-001311, piRNA-004153, piRNA-017723, piRNA-017724, and piRNA-019752	Illumina high-throughput sequencing (HTS)	220	220	Univariate and multivariate	Univariate analysis: piRNA-001311 HR = 0.626, piRNA-004153, HR = 1.048, piRNA-017723, HR = 0.769, piRNA-017724, HR = 0.231, and piRNA-019752, HR = 0.611	60	[13]
								Multivariate analysis: piRNA-017724, HR = 0.250		
7	Michael Bartos, et al (2021)	Czechia	piRNA-1849, piRNA-9491, piRNA-12487, piRNA-12488, and piRNA-23231	RNA sequencing	96	34	Not reported	HR for piRNA-23231 = -0.497	60	[45]
8	Xiaorong Zhou, et al (2020)	China	piRNA-1245	qRT-PCR	66	66	Univariate and multivariate	Univariate analysis: HR = 2.183	60	[46]
								Multivariate analysis: HR = 2.989		
9	Victor D Martinez, et al (2016)	Canada	FR222326	RNA sequencing	320	38	Not reported	HR = 10.22	100	[47]
10	Aswini R. Krishnan, et al (2016)	USA	NONHSAT123636, NONHSAT113708, NONHSAT067200, and NONHSAT081250	RNA sequencing	256	N/A	Not reported	HR for NONHSAT067200 = 1.897	134	[48]
11	Maarouf A Saad, et al (2019)	USA	piRNA-35373, piRNA-266308, piRNA-58510, and piRNA-38034,	RNA sequencing	40	N/A	Univariate and multivariate	Univariate analysis: piRNA-58510 HR = 0.072, and piRNA-35373 HR = 0.17	134	[49]
								Multivariate analysis: piR-58510 HR = 0.063, and piR-3537 HR = 0.089		
12	Chenming Zhao, et al (2019)	Germany	piRNA-34536, and piR-51810	qRT-PCR	148	90	Univariate and multivariate	Univariate analysis: piRNA-34536 HR = 0.295, and piRNA-51810 HR = 0.205	150	[50]
								Multivariate analysis: piRNA-34536 HR = 0.275, and piRNA-51810 HR = 0.223		
13	Dan Li, et.al (2016)	China	piRNA-651	qRT-PCR	78	N/A	Not reported	HR = 0.002	25	[25]
14	Preethi Krishnan, et al (2016)	Canada	piRNA-009051, and piRNA-021032^a^	RNA sequencing	104	11	Univariate and multivariate	Univariate analysis: HR = 2.31	200	[51]
								Multivariate analysis: HR = 2.29		
15	Anna Cordeiro, et al (2016)	Spain	piRNA-651	qRT-PCR	94	N/A	Not reported	HR = -1.297^a^	250	[52]
16	Wenhao Weng et al (2018)	USA	piRNA-1245	RNA sequencing	189	195	Univariate and multivariate	Univariate HR = 3.208	133	[53]
								Multivariate HR = 2.9347		
17	Junlan Feng et al (2020)	China	piRNA-823	Microarray analysis	176	N/A	Univariate and multivariate	Univariate HR = 7.49	150	[27]
								Multivariate HR = 8.02		
18	Dongmei Mai et al (2018)	China	piRNA-54265	qRT-PCR	535	N/A	Univariate and multivariate	Univariate HR = 2.02	70	[24]
								Multivariate HR = 1.99		
19	Natalie Firmino et al (2016)	Canada	FR018916, FR140858, FR197104, FR237180, and FR298757	RNA sequencing	455	43	not reported	FR018916, HR = 0.882, FR140858 HR = 1.000^b^, FR197104 HR = 0.091, FR237180 HR = 1.003, and FR298757 HR = 1.009	100	[54]
20	Wentao Zhang et al (2023)	China	piRNA-1742	RNA sequencing	96	N/A	not reported	Univariate analysis: piR-1742 HR = 2.232	120	[55]
								Multivariate analysis: piR-1742 HR = 1.993		

^a^In this study for both of piRNAs HR calculated as risk score

^b^The forest plot analysis does not take it into account due to its extremely low standard error (SE = 0)

**Table 2:** Main outcomes of the included studies

Authors	Cancer type	Sample type	Exploring the Influence of piRNA on biological outcomes
Huiying Han, et al	DLBCL	FFPE	Patients with a poor prognosis exhibited a significant increase in piRNA-30473 expression level in comparison to those with a favorable prognosis
Guoqing Li et al	OC	FFPE	High expression of piRNA-25783 in tumors is associated with shorter overall survival of patients compared to those with low expression
Lisha Ai, et al	MM	BM aspirates and PB	High expression of piRNA-piRNA-823 is correlated with reduced overall survival
Robert Iliev, et al	RCC	Tissue, Serum and Urine	High expression of piRNA-823 in tumor tissue is associated with unfavorable clinical outcomes
Jonas Busch, et al	RCC	Fresh-frozen tissues	The study found that increased levels of piRNA-30924 and piRNA-38756, and decreased levels of piRNA-57125 in metastatic primary tumors were strongly linked to tumor recurrence and shorter overall survival
Ailin Qu, et al	CRC	Serum	Low piRNA-017724 expression leads to lower OS and PFS rates compared to high expression
Michael Bartos, et al	GBM	Fresh-frozen tissues	Only piRNA-23231 was significantly decreased in GBM, which may play a role in GBM behavior and poorer survival. Additionally, piRNA-23231 shows promise as a potential prognostic biomarker
Xiaorong Zhou, et al	GC	GJ	High piRNA-1245 expression in gastric juice is associated with poorer OS and PFS in patients with gastric cancer
Victor D Martinez, et al	GC	Tissue	Only FR222326 was significantly associated with overall survival. Notably, lower expression levels of FR222326 were strongly correlated with a poorer prognosis in patients
Aswini R. Krishnan, et al	HNSCC	Tissue	The study found that NONHSAT067200, a piRNA that is downregulated in smoking-induced HNSCCs, was significantly associated with poor survival in patients
Maarouf A Saad, et al	HNSCC	Tissue	Low expression of piRNA-58510 and piRNA-35373 were found to significantly correlate with improved patient survival
Chenming Zhao, et al	RCC	Tissue and serum	piRNA-34536 and piRNA-51810 were found to be downregulated in ccRCC. Decreased tissue piRNA levels were found to be significant and independent predictors of shortened PFS, CSS, and OS in ccRCC patients
Dan Li, et al	NSCLC	Tissue	The study suggests that piRNA-651 functions as an oncogene in NSCLC, and that high expression levels of piRNA-651 are associated with a higher risk of death among patients with NSCLC
Preethi Krishnan, et al	BC	FFPE	The study found that hsa_piRNA_021032 was upregulated in tumor tissues and appeared to contribute to lower overall survival
Anna Cordeiro, et al	HL	FFPE	Patients who had low levels of piR-651 experienced a poorer outcome
Wenhao Weng et al	CRC	Tissue	Patients with high expression levels of piRNA-1245 demonstrated a significant inclination towards unfavorable overall survival outcomes
Junlan Feng et al	CRC	Tissue	Patients with CRC who overexpress piRNA-823 are more likely to experience unfavorable overall survival and exhibit a diminished response to adjuvant chemotherapy
Dongmei Mai et al	CRC	Tissue and serum	Levels of piRNA-54265 in CRC tissues were strongly correlated with the levels in the serum of patients. Furthermore, both tissue and serum levels of piRNA-54265 were found to be highly associated with clinical outcomes, including resistance to chemotherapy and poor survival in CRC patients
Natalie Firmino et al	HNSCC	Tissue	A group of patients with HPV-positive HNSCC who have a poor prognosis can be identified using a five-piRNA signature, which underscores the potential usefulness of piRNAs in managing patients
Wentao Zhang et al	RCC	Tissue	There is a significant association between high levels of piRNA-1742 expression in RCC samples and unfavorable clinical outcomes

*Abbreviations: OS* Overall Survival, *ISH* In situ hybridization, *FFPE* Formalin-Fixed Paraffin-Embedded, *BM* Bone Marrow, *PB* Peripheral blood, *GJ* Gastric juice, *GA* Gastric adenocarcinoma, *PFS* Progression-free survival, *CSS* Cancer-specific survival

### Mapping of piRNA(s) prognostic data

After full-text assessments, 20 studies meeting the search criteria were eligible for final review. An overview of the studies having eligible overall survival analysis data and their main clinical characteristics are shown in Tables 1 and 2.

Out of the 20 studies analyzed, four focused primarily on RCC [26, 44, 50, 55], four on CRC [13, 24, 27, 53], three on Head and neck squamous carcinoma (HNSCC) [48, 49, 54], two on Gastric cancer (GC) [46, 47], one on Breast cancer (BC) [51], one on Diffuse large B cell lymphoma (DLBCL) [41], one on Ovarian cancer (OC) [42], one on Multiple myeloma (MM) [43], one on Glioblastoma (GBM) [45], one on NSCLC [25], and one on Hodgkin lymphoma (HL) [52].

Out of these 20 studies, nine records utilized high-throughput assays of RNA sequencing [13, 45, 47–49, 51, 53–55], seven used qRT-PCR [24–26, 43, 46, 50, 52], three used Microarray analysis [27, 41, 44], and one used ISH [42] for quantification of piRNAs in patients biological specimen. The majority of these studies were conducted in China (nine studies) [13, 24, 25, 27, 41–43, 46, 55], three studies followed by the USA [48, 49, 53], three in Canada [47, 51, 54], two in Czech Republic [26, 45], two in Germany [44, 50], and one in Spain [52].

The biological samples collected for analysis included serum (*n* = 5) [13, 24, 26, 43, 50], tissue (*n* = 11) [24–27, 47–50, 53–55], Formalin-Fixed Paraffin-Embedded (FFPE) samples (*n* = 4) [41, 42, 51, 52], blood samples (*n* = 1) [43], fresh-frozen tissues (*n* = 2) [44, 45], gastric juice samples (*n* = 1) [46], bone marrow aspirates (*n* = 1) [43], and urine samples (*n* = 1) [26].

The selected articles were published between 2015 and 2023. The median follow-up time ranged from 25 to 250 months. We have summarized this information in Tables 1 and 2.

### Evaluation of the role of piRNA expression levels as potential prognostic markers in cancer patients

**Fig. 2:**
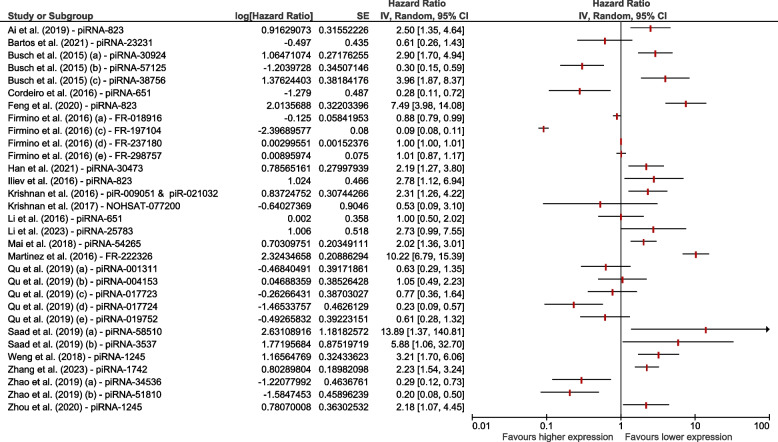
Forest plot on the association between the expression levels of different piRNAs and survival in patients with different types of cancer

We conducted an analysis of twenty eligible studies to investigate the role of piRNA expression levels as prognostic markers in different cancer patients. Our findings indicate that six piRNAs, including piRNA-57125 [44], piRNA-651 [25, 52], FR237180 [54], piRNA-017724 [13], piRNA-34536 [50], and piRNA-51810 [50], have a significant prognostic impact on survival prediction. Conversely, the expression levels of twelve different piRNAs, such as piRNA-823 [26, 27, 43], piRNA-1245 [46, 53], piRNA-30924 [44], piRNA-38756 [44], piRNA-30473 [41], (piRNA-009051 and piRNA-021032) [51], piRNA-54265 [24], piRNA-1742 [55], FR-222326 [47], piRNA-58510 [49], and piRNA-3537 [49], were found to be lower and associated with poor survival outcomes. It is important to note that only univariate OS analysis was conducted in this study which is illustrated in Fig. 2. Moreover, we refrained from performing a pooled analysis due to the potential for misleading interpretations when combining various piRNA data.

### The role of piRNA expression levels in the prognostic impact of renal cancer

**Fig. 3:**
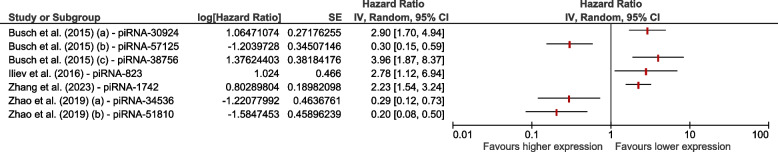
Forest plot on the association between the expression levels of different piRNAs and survival in patients with RCC

Figure 3 depicts the forest plot of HR for seven piRNAs studied across four records of patients with renal RCC [26, 44, 50, 55]. Our analysis revealed that higher expression levels of three piRNAs, namely piRNA-57125 [44], piRNA-51810 [50], and piRNA-34536 [50], were significantly associated with better OS in RCC patients. Conversely, the overexpression levels of four other piRNAs, including piRNA-823 [26], piRNA-1742 [55], piRNA-38756 [44], and piRNA-30924 [44], were significantly linked to poorer outcomes in patients with RCC.

### The role of piRNA expression levels in the prognostic impact of colorectal cancer

**Fig. 4:**
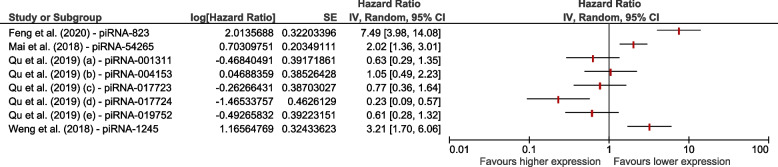
Forest plot on the association between the expression levels of different piRNAs on survival in patients with CRC

We also investigated the prognostic impact of piRNAs on survival prediction in CRC [13, 24, 27, 53] patients based on four records. We found that a higher expression level of only one piRNA, piRNA-017724 [13], was significantly associated with better survival and outcome. On the other hand, the higher expression levels of three different piRNAs: piRNA-823 [27], piRNA-54265 [24], and piRNA-1245 [53], were significantly associated with poorer survival outcomes (Fig. 4).

### The role of piRNA-823 expression as a prognostic and predictive marker for piRNA-mediated regulation in CRC, MM and RCC

**Fig. 5:**
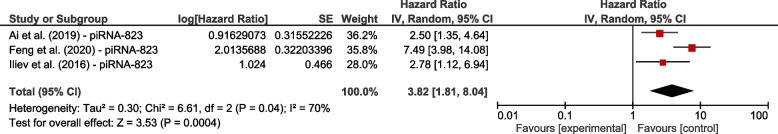
Forest plot on the association between piRNA-823 expression levels and survival outcome in CRC, MM and RCC patients

We performed a meta-analysis of three records in CRC [27], MM [43], and RCC [26] involving 836 patients to explore the association between overall survival and piRNA-823 expression. Pooled HRs showed that overexpression levels of piRNA-823 expression were associated with poorer OS (HR = 3.82; 95% CI, [1.81, 8.04]; *P* = 0.0004,) (Fig. 5). Our meta-analysis has attempted to combine multiple studies that are known to be heterogeneous in terms of cancer type. Our estimates of heterogeneity metrics have wide 95% CI indicating heterogeneity (I^2^ = 70%).

### Sensitivity analysis and publication bias

**Fig. 6:**
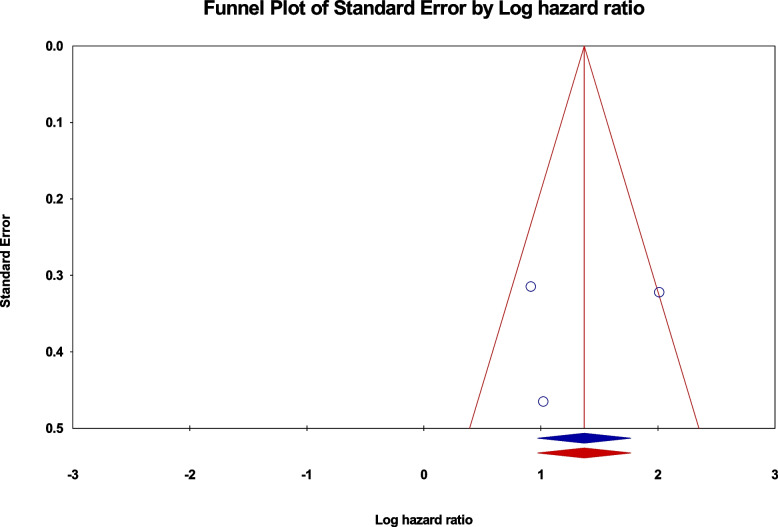
Funnel plot for the OS meta-analysis of piRNA-823 in different cancer types

Sensitivity analyses were conducted to investigate methodological heterogeneity in these studies and assess the impact of individual study data on the overall outcome. No significant influence of any single study on the overall outcome was identified (as shown in Fig. 6). Additionally, a potential publication bias was examined using the Funnel plot, revealing no apparent publication bias in the included studies.

## Discussion

Great improvements have been achieved in cancer detection and treatment. However, the 5-year survival rate remains relatively low for most cancers. Human health is seriously threatened by cancer [56]. piRNAs are a class of small non-coding RNA molecules that are expressed in the germline of many species [57]. They differ from other RNA molecules in their size and function [58]. Recent studies have suggested that piRNAs hold promise as biomarkers for early detection, prognosis, and novel biomarkers for progression and chemoresistance in cancer patients. Additionally, they may be useful in monitoring different cancer patients following treatment [59–62]. For example, the levels of piR-5937 and piR-28876 in the serum of CRC patients decreased significantly with the advanced clinical stage. However, both piRNA levels significantly increased in serum samples taken 1 month after surgery, indicating that their levels are linked to the presence of the tumor. These findings suggest that piRNAs could potentially serve as valuable tools for monitoring CRC progression and treatment response [63].

Recent research has highlighted inconsistencies in the role of piRNAs for monitoring patients, predicting relapse/recurrence, assessing treatment response, and determining patient prognosis. The potential role of piRNAs in contributing to survival prognosis remains uncertain. So, we collected different studies on piRNAs and how they affect the survival of cancer patients. Our goal is to do a meta-analysis to understand better how increased piRNA-823 expression relates to patient survival.

After conducting a systematic analysis of articles reporting on survival and piRNA data, we found that elevated expression levels of 12 specific piRNAs (piRNA-823, piRNA-1245, piRNA-30924, piRNA-38756, piRNA-30473, piRNA-009051, piRNA-021032, piRNA-54265, piRNA-1742, FR-222326, piRNA-58510, and piRNA-3537) are significantly associated with a higher risk of reduced OS in cancer patients. These piRNAs may play a proto-oncogenic role by promoting tumorigenicity when their expression is increased. Conversely, increased expression levels of 6 distinct piRNAs (piRNA-57125, piRNA-651, FR237180, piRNA-017724, piRNA-34536, and piRNA-51810) are significantly associated with improved OS in these patients. Increasing the expression of these piRNAs leads to decreased tumorigenicity.

Considering the larger amount of data for CRC and RCC compared to other cancer types, we concentrated our systematic analysis on these two types of cancer. This approach allowed us to conduct a more in-depth and comprehensive analysis of the available data, and to draw meaningful conclusions about the research question at hand. We acknowledge that this limitation may affect the generalizability of our findings to other types of cancer, and we have taken care to discuss this issue in the limitations section of our study.

The systematic analysis of data for RCC revealed that increasing the expression of three piRNAs (piRNA-57125, piRNA-51810, and piRNA-34536) is significantly associated with better OS. Conversely, increasing the expression of four different piRNAs (piRNA-823, piRNA-1742, piRNA-38756, and piRNA-30924) is significantly associated with decreasing OS. In the case of CRC, increased expression of piRNA-017724 is associated with increased OS in patients, while increased expression of three different piRNAs (piRNA-823, piRNA-54265, and piRNA-1245) is associated with decreased OS in patients. These findings suggest a crucial role for piRNAs in predicting patient outcomes in these specific cancer types.

We conducted a targeted meta-analysis on piRNA-823, given its significant role and the abundance of available records compared to other piRNAs. Our systematic review and meta-analysis revealed that overexpression levels of piRNA-823 are associated with poor prognosis across various types of cancer.

This meta-analysis included three studies that explained the functional roles of piRNA-823 in cancer prognosis. According to *Ai *et al., granulocytic-myeloid-derived suppressor cells (G-MDSCs) regulate cancer stemness in MM patients by activating piRNA-823, which leads to more DNA methylation and higher tumorigenic potential of MM cells [43]. *Liev *et al. demonstrated that piRNA-823 may play a role in preserving genomic stability, and the frequent loss of piRNA-823 in RCC tumor tissues could be associated with genomic instability, a common characteristic of malignant tumors They found that patients with RCC had significantly higher levels of piRNA-823 in their serum compared to healthy individuals. Furthermore, the up-regulation of piRNA-823 in the serum of RCC patients was associated with unfavorable clinical outcomes [26]. *Junlan Feng *et al. illustrated Knock-down of piRNA-823 inhibits the malignant characteristics of CRC cells and patients with CRC who have high levels of piRNA-823 expression are at a greater risk of experiencing poor OS and are less likely to respond positively to adjuvant chemotherapy [27].

Our findings suggest that seven specific piRNAs in RCC and four piRNAs in CRC could be useful for monitoring cancer patients and conducting survival analysis.

We suggest creating a test kit for piRNA-823 to assess the prognosis and monitoring of cancer patients. Additionally, we propose the creation of a panel consisting of 18 piRNAs for follow-up and treatment monitoring of cancer patients. Implementing these recommendations has the potential to improve patient outcomes and enhance the efficacy of cancer management strategies.

### Strengths

Our meta-analysis offers several advantages. 1) It is the first study to systematically evaluate the correlation between piRNA expression and survival outcomes in cancer patients. 2) Our findings hold significant value, indicating that the sample size included in this meta-analysis was adequate. 3) This study showed high expression levels of piRNA-823 expression in cancer patients cause a greater risk of experiencing poor OS. 4) The included studies varied in the cancer pathological type that were analyzed, which often resulted in substantial heterogeneity between studies in the strength of the predictive effect.

### Limitations

Although our meta-analysis has several strengths, it is important to acknowledge some of its limitations, which include: 1) This meta-analysis was limited to the evaluation of univariate OS due to the available studies that could be included in the analysis. 2) Survival analysis requires suitable data for PFS, DFS, and RFS. However, the lack of such data poses a significant challenge in this regard. 3) Obtaining raw data is crucial for survival prediction and analysis. However, the lack of access to such data poses a significant challenge, and researchers often have to rely on data obtained from the K-M chart. 4) The insufficiency of adequate data has resulted in the absence of meta-analyses on other piRNAs in various types of cancers. 5) Due to the potential for misleading interpretations when combining various piRNA data, a pooled analysis was not performed.

We detected 33 piRNAs in this study, mostly in serum or plasma. This may be because blood samples from cancer patients are more accessible. On the other hand, saliva, urine, and other body fluids are seldom used in research.

## Conclusion

In conclusion, we have gathered a substantial amount of prognostic data on the association of various piRNAs with survival. This meta-analysis and systematic review provided evidence of a correlation between dysregulated piRNA expression levels and survival outcomes in patients with different types of cancer. Our findings indicated that altered expression of piRNA-823 was significantly associated with survival outcomes in cancer patients. Overexpression of piRNA-823 was linked to decreased OS in various cancer patients, suggesting its potential as a promising biomarker for predicting the prognosis of human cancers. In the future, further large-scale studies should be conducted to verify the clinical applications of altered piRNAs in assessing the prognosis of different cancers.

## Data Availability

Not applicable.
